# Lifestyle interventions for dementia risk reduction: A review on the role of physical activity and diet in Western and Asian Countries

**DOI:** 10.1016/j.tjpad.2024.100028

**Published:** 2025-01-01

**Authors:** Amelia Nur Vidyanti, Fitri Rahmawati, Rifki Habibi Rahman, Astuti Prodjohardjono, Abdul Gofir

**Affiliations:** a Department of Neurology, Faculty of Medicine, Public Health, and Nursing, Universitas Gadjah Mada, Indonesia/Dr Sardjto General Hospital Yogyakarta, Indonesia; b Neurology Research Office, Department of Neurology, Faculty of Medicine, Public Health, and Nursing, Universitas Gadjah Mada, Indonesia

**Keywords:** Dementia risk reduction, Physical activity, Dietary intervention, Cognitive health, Western countries, Asian countries

## Abstract

Dementia, is a critical global public health challenge with no effective pharmacological treatments. Recent research highlights the significant role of lifestyle interventions, particularly physical activity and dietary habits, in mitigating cognitive decline among the elderly and preventing the progression to dementia in individuals with Mild Cognitive Impairment (MCI). This comprehensive review explores the impact of physical exercise and dietary approaches on cognitive health, comparing strategies adopted in Western and Asian countries. Physical activity, including aerobic, resistance, balance training, and dual-task exercises, has been shown to enhance neurogenesis, improve cerebral blood flow, and delay cognitive decline. In Western countries, structured regimens such as the Mediterranean (MedDiet) and MIND diets are prominent, while Asian countries often integrate traditional mind-body practices like Tai Chi and culturally relevant diets rich in antioxidants and polyphenols. Although both regions recognize the importance of lifestyle changes in reducing dementia risk, their approaches differ significantly, shaped by cultural norms and dietary preferences. This review underscores the need for culturally tailored public health strategies to promote cognitive health globally, highlighting the importance of individualized approaches in MCI and dementia prevention.

## Highlights

• Physical exercise and diet are critical components in reducing the risk of dementia.
• Although Western and Asian countries recognize the importance of physical exercise and diet in dementia risk reduction, their approaches and cultural practices may differ significantly.
• Western countries tend to emphasize structured exercise regimens and specific dietary patterns like the Meddiet and MIND diet.
• Asian countries integrate traditional mind-body exercises and culturally relevant dietary practices, highlighting the role of holistic and culturally tailored interventions in promoting cognitive health.

## Graphical abstract

**Figure:**
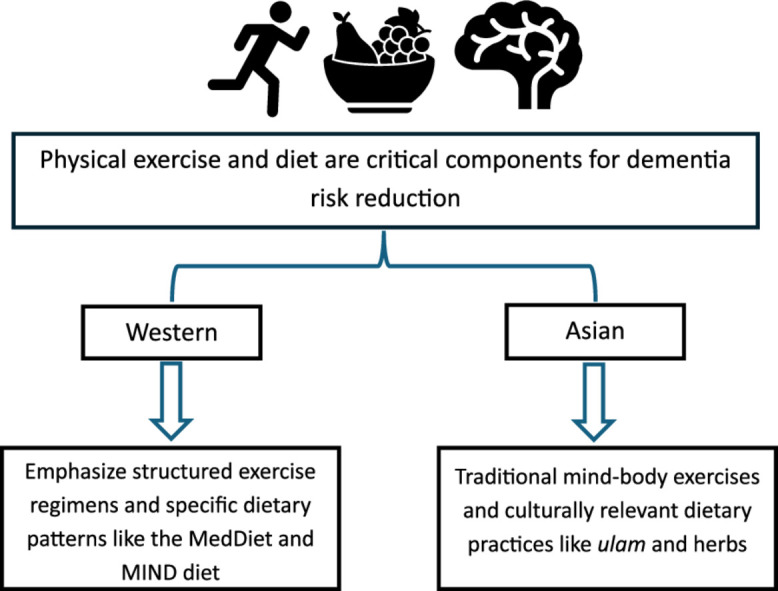

## 1 Introduction

Dementia, characterized by a decline in cognitive function severe enough to interfere with daily life, is a growing public health concern worldwide [1]. Alzheimer's disease is the most common form of dementia. To date, there are no pharmacological treatments that can cure cognitive impairment and dementia [1]. Current research underscores the potential of lifestyle interventions, particularly physical exercise and dietary habits, in reducing the risk of dementia [2,3]. However, the effectiveness and adoption of these interventions can vary significantly between Western and Asian countries due to cultural, dietary, and lifestyle differences. This review aims to highlight the differences in physical exercise and dietary preferences between Western and Asian countries in dementia risk reduction strategies, and to provide further recommendations.

## 2 Physical activity, regular exercise, and brain health

Physically active individuals tend to have a lower risk of cognitive impairment or dementia compared to those who are inactive or sedentary [4,5]. Participation in regular exercise and physical activity may improve cardiovascular fitness, increase blood flow to the brain [6], support neurogenesis, synaptogenesis, and neuroplasticity through induction of neuron to produce Brain-derived Neurotrophic Factor (BDNF) [7,8], reduce neuronal loss, as well as positively impacts the brain aging process and is associated with greater brain volume in areas vulnerable to dementia [9], such as the hippocampus, temporal [10], and frontal regions [5,11,12].

Physical activity shows significant influence of brain circuit through alteration of Functional Brain Network Connectivity (FBNC), where brain neurons are separated by its location but the interconnection among them still be synched [7]. Physical activity can also induce regulation of PGC-1alpha which is a central regulator of metabolism that can modify multiple level in gene expression [13]. In hippocampal region, PGC-1alpha can induce formation of dendritic spines along with co-regulatory substance like BDNF [14]. Moreover, other studies revealed that physical activity may induce the production of lactate which can promote myelination and memory formation by breakdown of glycogen in neuron which enhance transport of neurotransmitter [8,14]. All of these could reduce the risk of dementia and cognitive decline.

In addition to maintaining cognitive health, daily physical activity can help delay dependency in people with dementia by reducing the risk of poor age-related physical health outcomes such as falls, muscle and bone injuries, and disability [15].

Conversely, lack of physical activity is a global issue and has been identified as one of the twelve modifiable risk factors in the development of dementia [2,16]. Exercising and being physically active regularly at all stages of life is beneficial even in old age and has been shown to provide neurological protection by reducing the risk of dementia by 30% to 45%, particularly for Alzheimer's disease [2,17].

As a lifestyle intervention, exercise and physical activity, regardless of type, have been shown to reduce the risk of dementia within just 6–8 weeks [18,19]. However, a pragmatic approach is needed to encourage older adults, whether in Western or Asian countries and regardless of cognitive impairment, to participate in regular exercise and physical activity. Therefore, alternative options for participating in an equivalent amount of exercise and physical activity over a week, regardless of frequency, can help older adults achieve their weekly physical activity targets [20]. Shorter but more frequent exercise sessions can provide greater cognitive benefits for older adults with mild cognitive impairment [21].

Western and Asian populations showed differences in baseline metabolic activity, which can modify the impact of physical activity among these groups. Previous research has demonstrated that ethnic differences can influence visceral adiposity, insulin resistance, and impaired insulin secretion [22]. These metabolic disparities can, in turn, affect physical fitness during exercise for both populations. Ethnicity impacts insulin resistance due to various genetic factors that influence the sensitivity of pancreatic beta cells [23,24]. Additionally, population studies have shown that Asians generally have a higher body fat composition than Western populations, even when matched for Body Mass Index (BMI) [25,26].

### 2.1 Physical exercise in western countries

Physical exercise is widely recognized and promoted as a vital component of a healthy lifestyle [20]. Compared to Asian Countries, Western populations genetically have higher cardiorespiratory fitness [27]. It assumed that the Western populations have higher mitochondrial activity than Asian. Previous studies showed that with the same type of physical activity, different ethnical have different cardiorespiratory fitness due to VO2 max prediction values [28]. In Western countries, physical exercises that have been recommended for reducing the risk of dementia are regular aerobic, resistance training, balance exercise, and dual-task training. Regular aerobic activities such as running, jogging, brisk walking, cycling, and swimming are commonly recommended for maintaining cardiovascular and cognitive health [29]. Resistance training and balance exercises are also emphasized to improve muscle strength and prevent falls, which are critical for the aging population [30]. The cultural emphasis on structured exercise regimens and gym-based workouts supports these recommendations, with substantial evidence showing that physically active individuals have a lower risk of cognitive impairment and dementia [10].

#### 2.1.1 Aerobic exercise

Aerobic exercise is crucial for improving, maintaining, or preserving cognitive function in older adults, as various studies highlight its benefits [31]. Aerobically trained older adults may have better cerebrovascular function, showing a link between aerobic fitness, cerebrovascular function, and cognitive function [32,33]. Aerobic exercise also enhances cerebral function by modulating cardiovascular markers, reducing total cholesterol, and increasing peak oxygen uptake, all of which contribute to better cardiovascular health and endothelial function [33]. Research also indicates increased brain volume and cognitive function resulting from aerobic exercise in older adults [31]. Improved cerebrovascular function can even occur in older adults already diagnosed with cerebrovascular dysfunction, making it an effective management intervention, not just a preventive measure [32,34].

#### 2.1.2 Resistance or strength training

Resistance or strength training involving various equipment has been shown to improve global cognition and executive function in older adults and enhance their ability to perform daily activities [35,36]. This type of training induces the release of more growth factors and neurotrophic factors that support exercise-induced cognitive improvements [35].

#### 2.1.3 Balance training

Maintaining balance while aging is crucial for performing daily activities. Functional independence can reduce motor and cognitive dysfunction during multitasking that may impair balance performance [37]. Impaired brain health, especially white matter lesions [38], makes individuals with cognitive impairment more prone to falls compared to those without cognitive impairment [39]. The risk of falls increases when individuals with MCI face cognitive challenges such as dual-tasking common in functional daily activities [40]. Balance training stimulates the vestibular, neuromuscular, and proprioceptive systems, leading to connections between the vestibular nucleus and the cerebellum, hippocampus, and prefrontal and parietal cortex, enhancing brain flexibility and motor function [41,42]. Therefore, balance training should be conducted carefully in a safe environment to prevent accidental falls. Conventional balance exercises include tandem walking, single-leg standing, weight shifting, slalom walking, turning, standing or walking on heels or toes, and spinning. Balance training can be intensified gradually, such as doing it with eyes closed or on unstable surfaces like foam pads [30].

#### 2.1.4 Dual task training (DTT)

Dual task training involves performing cognitive and motor tasks simultaneously [43]. DTT has the potential to enhance cognitive and motor functions, such as global cognition, executive function, memory, mobility, strength, and balance in older adults [42,44,45]. DTT involves a combination of motor tasks like walking while carrying objects or manipulating objects (e.g., buttoning and unbuttoning a shirt, zipping and unzipping, or tying a thread) and cognitive tasks like spelling, doing arithmetic without a pencil and paper, or naming objects verbally [42]. Besides multitasking, DTT also considers multisensory elements that provide additional cognitive load and improve cognitive capacity through repetition [41].

### 2.2 Physical exercise in Asian countries

Other than recommended regular exercise, traditional physical activities are often integrated into daily life rather than structured exercise routines in many Asian countries. Practices such as Tai Chi and yoga, which emphasize the exercise for mind-body connection, are prevalent and culturally accepted [46]. These exercises involve gentle movements, offering benefits for balance, flexibility, and cognitive health [47]. Studies suggest that these traditional exercises not only improve physical health but also enhance cognitive functions, making them effective non-pharmacological interventions for dementia prevention in Asian populations. The cultural relevance and holistic approach of these practices contribute to their widespread adoption and effectiveness [48].Traditional Chinese exercises (TCE) like Tai Chi and yoga are other effective approaches for promoting balance and cognitive health. These exercises emphasize the mind-body connection and are performed calmly, consisting of gentle movements, physical postures, breathing techniques, and meditation [[49], [50], [51]]. Mind-body exercises are meditative and support social aspects [52]. Evidence suggests that yoga and TCE target aerobic function, flexibility, lower bo[dy strength, balance, coordination, cognition, perception, executive function, processing speed, and attention while reducing stress [53] and improving cognitive domains such as memory, language ability, visual perception, and sleep quality in adults [50,[54], [55], [56], [57], [58]]. Furthermore, TCE is safe and recommended as physical activity, especially in East and Southeast Asia, where most research has been conducted [59]. In the Asian context, mind-body exercises may be considered more culturally relevant [60].

### 2.3 Multicomponent exercise

Multicomponent exercise combines at least two different types of exercise, including aerobic, resistance (strength), and balance, as well as mind-body exercises and dual task training [32]. In older adults with cognitive impairment, multicomponent exercise has been shown to provide better cognitive benefits than single exercises, particularly in terms of global cognition [[61], [62], [63], [64], [65], [66]]. Additionally, chair-based multicomponent exercises effectively engage older adults with walking difficulties or disabilities in physical activity, providing positive impacts on cognition, physical function, and psychosocial aspects [67].

### 2.4 Recommended strategies for exercise and physical activity in diverse population (Western and Asian countries)

1. Older adults are recommended to engage in 150–300 min of moderate-intensity aerobic exercise or physical activity per week, or 75–150 min of vigorous-intensity aerobic exercise or physical activity per week, or a comparable mix of both moderate and vigorous activities [68].
2. Strength, balance, and/or multicomponent exercises should be performed at least twice a week. Older adults can start with a tolerable amount of exercise that involves multiple enjoyable components, performed individually or in groups, and gradually increase the exercise volume over time to achieve cognitive benefits from physical activity [69].
3. Traditional mind-body exercises like Tai Chi and yoga are safe and effective alternatives, especially for those with a preference for Asian cultural practices [59].
4. For older adults who may not be able to start moderate-intensity exercise due to various constraints such as physical impairment, disability, poor endurance, functional capacity, or low motivation, engaging in low-intensity exercise can also yield positive effects on cognition [70,71].
5. Conducting shorter but more frequent exercise sessions over time can provide greater cognitive benefits for elderly with MCI [21].

## 3 Nutrition, diet, and dementia

Dietary modifications and/or nutritional supplementation are attractive options for preventing and treating age-related cognitive decline and dementia. The World Health Organization (WHO) supports dietary changes as part of lifestyle interventions aimed at lowering the risk of cognitive decline or dementia in older adults [1]. Although the Lancet commission on dementia prevention, intervention, and care does not list diet as a major contributor to dementia, it should be noted that risk factors for diseases such as obesity, hypertension, diabetes, and a combination of cardiovascular risk factors are associated with diet as a major contributor [2]. Studies in the Netherlands, such as the Rotterdam study, report a relationship between diet quality and greater brain tissue volume, which may indicate that nutrition plays a role in combating neurodegeneration through brain structure like white matter [73]. Based on existing evidence, dietary approaches appear to be one of the most effective strategies in preventing cognitive decline and dementia in older adults.

Beyond cultural aspect, different effect of diet mainly influenced by the food compositions among Asian and Western populations. Asian local foods have more total fat and energy than Western food [74]. Difference of food composition between Asian and Western might also being influenced by the topography of each country. Regional food preferences significantly impact environmental sustainability, with Asian diets maintaining lower animal product consumption compared to Western diets [75].

### 3.1 Nutrition and diet in Western countries

Western dietary patterns, particularly the Mediterranean diet (MedDiet), have been extensively studied for their role in reducing dementia risk. known for its emphasis on vegetables, legumes, fruits, nuts, olive oil, and fish, while limiting red meat and saturated fats, is linked to enhanced cognitive function and lower levels of inflammatory markers by maintaining a balance between reactive oxygen species (ROS) and antioxidant compounds [76]. Western diet with those ingredients contain many substance to alter transcription trough non-coding RNAs and some transcription factor [77]. Additionally, the MIND diet, which combines elements of the MedDiet with specific recommendations to support brain health, has shown significant promise in slowing cognitive decline [78]. However, adherence to these diets can be challenging due to the availability and cultural preferences for processed and fast foods high in saturated fats and sugars.

#### 3.1.1 Mediterranean diet (MedDiet)

The Mediterranean diet (MedDiet) is characterized by high consumption of vegetables, legumes, fruits, nuts, olive oil, and fish, while reducing the intake of red meat, dairy products, and saturated fats [79]. Adherence to this dietary pattern can influence the composition of specific gut microbiota associated with reduced physical frailty, improved cognitive function, and decreased inflammatory markers such as C-reactive protein and interleukin-17 [80]. Therefore, the NU-AGE diet, specifically designed to meet the dietary needs of older adults, has been shown to slow age-related cognitive decline after one year in older adults with or without mild cognitive impairment in a randomized controlled trial (RCT) [81]. Recently, the Green-MED diet, rich in polyphenol foods and low in red and processed meat, has been shown in RCTs to amplify the positive effects of the traditional MedDiet and potentially act as a brain-protective measure against aging-related atrophy [82].

#### 3.1.2 MIND diet

The MIND diet suggests 10 brain-healthy food groups (such as leafy green vegetables) and avoids five unhealthy food groups (such as red meat and its products, butter/margarine, cheese, pastries and sweets, and fast food) [83]. A systematic review by Kheirouri and Alizadeh [78] shows that adherence to the MIND diet positively correlates with specific cognitive domains and overall cognitive function in older adults [78]. The review highlighted that cognitive decline rates varied between 77% and 19% for each one-point rise in the MIND diet score across different studies. Adjibade et al. [84] found that a one-point increase in the MIND diet score was linked to a lower risk of subjective memory complaints [84].

#### 3.1.3 Ketogenic diet and beverages

The ketogenic diet is a high-fat, low-carbohydrate diet that induces a metabolic effect similar to starvation by forcing the body to use fat as the primary fuel source. A review suggests that the ketogenic diet has the potential to treat or prevent the progression of dementia [85]. Ketone bodies alter neuronal and astrocyte bioenergetic infrastructures, mediate glia-neuron interactions, impact energy balance, modify gene expression, post-translate proteins, and operate as signalling molecules [[86], [87], [88], [89]]. However, the difficulty in adhering to dietary changes among older participants with cognitive impairment may outweigh the benefits at more advanced stages of the disease [90]. Therefore, an RCT introduced the use of ketogenic beverages to improve a significant portion of brain glucose deficits and improve some cognitive outcomes in MCI with a dose of 30 g/day for 6 months [91]. However, most participants reported gastrointestinal side effects during the project, suggesting that tolerance to ketogenic beverages should be considered, especially among older participants [92].

#### 3.1.4 Caloric restriction and intermittent fasting

Western studies define caloric restriction (CR) as reducing daily caloric intake by 20–50% while maintaining the intake of essential nutrients to ensure health without causing malnutrition [93]. Although CR has positive impacts on brain health, this strategy is challenging to maintain over time and can negatively impact muscle mass [[94], [95], [96]]. Several human observational studies suggest that intermittent fasting (IF), without changes in calorie or nutrient intake, can positively impact cognition and brain health by increasing brain-derived neurotrophic factor (BDNF) expression, stimulating neurogenesis, and improving knowledge acquisition and retention [[97], [98], [99]]. In Tunisia, adults who remained physically active at least three times a week during Ramadan were able to improve their cognitive performance, suggesting that consistent physical activity practice combined with fasting can balance potential negative effects on cognitive performance [100].

#### 3.1.5 Omega-3 fatty acids

Diet with high Poly-unsaturated Fatty Acid (PUFA) can influence white matter integrity in brain by increasing synaptogenesis, increasing axonal and neuronal membrane cell integrity [101,102]. PUFA like Omega-3 and Omega-6, can act as a anti-oxidant for reducing Reactive Oxygen Species (ROS) and Reactive Nitrogen Species (RNS) thus can prevent aging in cell [103]. Studies about the potential role of omega-3-fatty acids in improving cognitive function mostly originate from Western countries. A systematic review highlighted the potential of omega-3 supplementation as a preventive or therapeutic tool for cognitive decline in older adults [104].

A specific combination of high-dose antioxidant vitamins, omega-3, and omega-6 has been shown to improve cognitive function and functional capacity in older adults with MCI [105]. Supplementation for 24 months with 1 g of fish oil (with 430 mg docosahexaenoic acid, 90 mg eicosapentaenoic acid), 22 mg carotenoids (10 mg lutein, 10 mg meso‑zeaxanthin, 2 mg zeaxanthin), and 15 mg vitamin E (D-alpha-tocopherol) has demonstrated improved cognitive performance in older adults, particularly in working memory [106]. These findings underscore the importance of nutritional enrichment in enhancing cognition and highlight how omega-3 combinations with other nutritional components can further reduce cognitive decline and delay the onset of dementia in old age

### 3.2 Nutrition and diet in Asian countries

In Asian countries, dietary habits are often centered around traditional foods rich in antioxidants and polyphenols, which are beneficial for cognitive health [107]. For instance, the Chinese MIND diet (CMIND) adapts the principles of the MIND diet to include culturally specific foods such as mushrooms, seaweed, garlic, and tea, while replacing less common ingredients like olive oil with vegetable oil [108]. Traditional diets in Asia, including the high consumption of fruits, vegetables, and fish, and the use of herbs and spices like turmeric (rich in curcumin), have been linked to lower dementia risk [109]. Furthermore, unique dietary practices such as the consumption of tropical *ulam* and probiotics from fermented foods are integral to the Asian diet and contribute to cognitive health through their anti-inflammatory and gut-brain axis effects [110].

#### 3.2.1 Chinese MIND diet (CMIND)

The Chinese MIND diet (CMIND) was developed based on the main principles of the MIND diet to meet the dietary characteristics of older Chinese individuals. In the CMIND diet, mushrooms/seaweed and garlic were added. Since olive oil is rarely used in China, vegetable oil is recommended as a replacement due to its higher unsaturated fatty acid content. Grapes were also replaced with tea. However, since butter/margarine and cheese are rarely consumed by older Chinese individuals, they were excluded from the CMIND diet. Red meat was also excluded as it is not recommended in the Chinese Dietary Guidelines [108]. A cross-sectional study conducted by Huang et al. [108] reported that older Chinese individuals with moderate and high adherence to the CMIND diet had lower odds of cognitive impairment and improved cognitive function [108]. Additionally, adherence to the Japanese diet, which shares similar components with the MedDiet, such as higher consumption of fruits, vegetables, and fish and lower consumption of red meat, was associated with a reduced risk of dementia incidence [111].

#### 3.2.2 Caloric restriction and intermittent fasting

Studies about the role of caloric restriction and intermittent fasting (IF) in Asian countries are scarce. A large cohort study conducted among older adults in Malaysia showed that IF has also been noted as one of the factors reducing the risk of cognitive impairment [112]. Additionally, another cohort study among people with mild cognitive impairment (MCI) in Malaysia revealed that IF regulates cognitive function through various metabolite pathways, including ketone body synthesis and degradation, butane metabolism, pyruvate metabolism, and glycolysis and gluconeogenesis pathways [113].

#### 3.2.3 Fruits and vegetables

To understand the impact of dietary interventions on cognitive function in old age, focus has been given to individual components, primarily due to their high antioxidant or polyphenol content. A recent meta-analysis found that increased daily fruit and vegetable intake was significantly associated with reduced cognitive impairment in older adults, possibly due to their high polyphenol and phytoestrogen content [114]. In an observational study, Rivan et al. [115] also stated that the abundant consumption of tropical fruits acts as a protective factor against dementia risk in older adults in Malaysia [115]. A previous RCT on tropical fruit juice TP 3-in-1 rich in polyphenols in 31 middle-aged women in Malaysia showed that consuming 500 ml of the beverage three times a day for ten weeks improved cognitive function, particularly in terms of learning, memory, information processing, mental flexibility, and visual-motor skills [116].

#### 3.2.4 Ulam consumption

In Asia, tropical *ulam* has long been consumed for its health benefits. These *ulams* are rich in polyphenols and antioxidants. The five most common *ulams* are *petai* (Parkia speciosa), *pucuk paku* (Diplazium esculentum), *ulam raja* (Cosmos caudatus), *pegagan* (Centella asiatica), and *kesum* (Polygonum minus) [110,117]. Several RCTs have been conducted on older adults to evaluate the effects of *ulam* on cognition and dementia prevention. Supplementation of Polygonum minus extract for 6 weeks at a dose of 250 mg (2 capsules/day) improved attention, short-term memory, and quality of life in social functioning in middle-aged women [118]. Supplementation of Cosmos caudatus for 12 weeks at a dose of 500 mg/day has the potential to improve global cognitive function as assessed by neuropsychological tests and brain imaging, possibly by reducing lipid peroxidation and increasing serum glutathione levels [119]. Both studies reported no harmful or toxic effects on health; however, these trials involved small sample sizes and short study durations.

#### 3.2.5 Herbs and spices

Asian countries have various herbs and spices and some of them have been investigated for their potential roles in reducing cognitive decline. Curcumin has been intensively studied for its neuroprotective potential. In observational cohort studies, consumption of curcumin-rich foods such as curry was associated with higher maintenance of cognitive function, particularly in attention, short-term memory, visual-spatial construction, language, and executive function in older adults in Singapore [120]. A review indicates that curcumin could be a promising therapy for addressing cognitive decline by reducing oxidative stress, systemic inflammation, and inhibiting pathways that reinforce these processes by modulating the activity of NOD-like receptor pyrin domain-containing-3 (NLRP3) inflammasome that responsible in some neuroinflammation pathology in human brain [[121], [122], [123], [124], [125]]. Curcumin may help break ROS that influence neuroinflammation and slows the progression of neurodegenerative diseases by reducing cell apoptosis, lowering lipid peroxidation, and increasing the activity of several antioxidant enzymes, such as glutathione and superoxide dismutase (SOD) [126,127].

Additionally, the continued consumption of Hericium Erinaceus (HE), a type of mushroom, is a convenient method for preventing dementia. Various chemical compounds, including hericenones in mushrooms, have dual effects on brain nerve tissues and improve cognition in older adults in Japan [128].

A systematic review shows that green tea consumption can reduce the risk of dementia, Alzheimer's disease, mild cognitive impairment, or cognitive impairment through several mechanisms, including antioxidant activity (e.g., catechins and polyphenols), anti-inflammatory effects, inhibition of amyloid-beta aggregation, and maintaining healthy blood vessels [129]. A special type of green tea, matcha, contains higher nutrient levels than regular green tea, particularly vitamin K and lutein, which both have beneficial effects on cognitive function in older adults [130,131]. These findings are supported by an RCT which reported that daily supplementation with matcha green tea powder (3 g/day) significantly improved cognitive function, particularly in the language domain [132].

#### 3.2.6 Probiotics

Probiotics, live microorganisms that confer health benefits to the host when administered in adequate amounts, have gained significant attention for their potential role in reducing the risk of dementia [133]. The gut-brain axis, the bidirectional communication pathway between the gut microbiota and the brain, is a key mechanism through which probiotics exert their effects on cognitive health. Probiotic bacteria have been shown to affect the dynamics and homeostasis of gut microbes, influencing gut and distal organ physiology, including the brain by producing Short Chain Fatty Acid (SCFA) which regulate gene expression by epigenetic mechanisms [134] The level of neurotransmitters and neurotrophic factors may be modulated by SCFA which subsequently. Moreover, SCFAs can disrupt the protein-protein interactions between amyloid-β (Aβ) peptides, hindering their formation into neurotoxic oligomers, which are the primary agents causing synaptic dysfunction and cognitive impairments in Alzheimer's disease. This would eventually lead to cognitive decline [135].

Studies using probiotics in people with cognitive impairment predominantly originate from Asian countries. In Western countries, the consumption of probiotics typically comes from supplements and fortified foods such as yogurt, kefir, and other dairy products [136]. There is a greater reliance on scientifically formulated probiotics and a trend toward choosing convenience-focused products like pills and powders over traditional fermented foods. Probiotics are accepted as a concept, legislated by health claims with clinically tested [137].

In Asian countries, there is a long history of consuming probiotics naturally through fermented foods, which are integral to their traditional diets. Fermented foods such as kimchi, natto, miso, tempeh and various pickled vegetables are staple components of Asian diets. These foods are rich in naturally occurring probiotics and are consumed regularly, providing continuous and diverse probiotic intake [136]. Asian fermented foods may contain a more diverse range of probiotics due to natural fermentation processes and the focus is often broader than just gut health, including longevity, and disease prevention [136].

Probiotic intervention for 12 weeks with 4 capsules per day (1 × 10^9 CFU of Bifidobacterium bifidum BGN4 and Bifidobacterium longum BORI in soybean oil) provided benefits on serum BDNF levels through interactions between gut microbiota and host BDNF, thereby improving brain function in older populations [138]. Similarly, other studies using *Bifidobacterium breve* A1 (MCC1274) showed that the consumption of this probiotic strain significantly improved cognitive function after 16–24 weeks of intervention in the MCI population [139,140]. These studies provide evidence that probiotic supplementation benefits cognitive and mental health in older adults living in the community with changes in gut microbiota composition. However, these studies involved relatively small sample sizes and short study periods (Table 1).

**Table 1:** Summary of evidence-based FITT recommendations for strategies to increase physical activity levels in older adults to reduce the risk of cognitive decline and dementia in Western and Asian population [47,72].

Exercise	Frequency	Intensity	Duration/Time	Type
Aerobic	3–7 days/week	Moderate to very high	150–300 min/week	Running, jogging, brisk walking, cycling, swimming, aerobic dancing
Strength	2–3 times/week	Moderate to high	8–12 repetitions/exercise, 1–3 sets (as tolerated)	Working on 2–3 muscle groups per session. Examples: Free weights, body weight, resistance bands, exercise machines
Balance	2–3 times/week	Light to moderate, gradually increased as tolerated	20–90 min/session	Balance exercises, dual task training
Mind-body exercise/Tai Chi	2–3 times/week	Light to moderate	20–60 min/session	Physical postures, breathing techniques, and meditation
Multicomponent	2 times/week	Moderate to very high	60 min/session	Combination of aerobic, strength, and/or balance exercises. Optimally in group settings

### 3.3 Recommendation strategies of nutrient and dementia in diverse population (Western and Asian countries)

It is recommended to adopt a healthy and balanced diet according to WHO [1] guidelines.

1. Diets resembling the Mediterranean or MIND diet can be integrated into daily eating habits to reduce the risk of cognitive decline and/or dementia.
2. Increasing dietary variety by consuming a diverse range of foods, including fruits, vegetables, *ulam*, nuts, mushrooms, probiotics, and green tea.
3. It is advisable to seek dietary and nutritional counselling to receive support and guidance in following a healthy and balanced diet.

## 4 Conclusion

The comprehensive review highlight that physical activity and diet are critical components in reducing the risk of cognitive decline and preventing dementia in both Western and Asian populations. Regular physical activity promotes neurogenesis, enhances cerebral blood flow, and reduces inflammation and oxidative stress. Concurrently, a diet rich in antioxidants, anti-inflammatory agents, and nutrient-dense foods supports brain health through multiple pathways. While both Western and Asian countries recognize the importance of physical exercise and diet in dementia risk reduction, their approaches and cultural practices differ significantly. Western countries tend to emphasize structured exercise regimens and specific dietary patterns like the MedDiet and MIND diet. In contrast, Asian countries integrate traditional mind-body exercises and culturally relevant dietary practices, highlighting the role of holistic and culturally tailored interventions in promoting cognitive health. Understanding these differences is crucial for developing effective, culturally sensitive public health strategies to combat dementia globally.

## CRediT authorship contribution statement

**Amelia Nur Vidyanti:** Writing – review & editing, Writing – original draft, Validation, Resources, Methodology, Investigation, Data curation, Conceptualization. **Fitri Rahmawati:** Writing – original draft, Visualization, Project administration. **Rifki Habibi Rahman:** Writing – review & editing, Project administration. **Astuti Prodjohardjono:** Writing – review & editing, Supervision, Investigation. **Abdul Gofir:** Writing – review & editing, Supervision, Investigation.

## Conflict of interest

The authors have no conflict of interest to report.
